# Physiologic Electrical Fields Direct Retinal Ganglion Cell Axon Growth In Vitro

**DOI:** 10.1167/iovs.18-25118

**Published:** 2019-08

**Authors:** Kimberly K. Gokoffski, Xingyuan Jia, Daniel Shvarts, Guohua Xia, Min Zhao

**Affiliations:** 1 Roski Eye Institute, University of Southern California, Los Angeles, California, United States; 2 Department of Ophthalmology and Vision Sciences, University of California, Davis, Sacramento, California, United States; 3 Medical Research Center, Beijing Chaoyang Hospital, Capital Medical University, Beijing, China; 4 Department of Dermatology, Institute for Regenerative Cures, University of California, Davis, Sacramento, California, United States; 5 Department of Psychiatry and Behavioral Sciences, University of California, Davis, Sacramento, California, United States

**Keywords:** retinal ganglion cell, axon guidance, optic nerve regeneration, electrical field

## Abstract

**Purpose:**

The purpose of this study was to characterize the ability of applied electrical fields (EFs) to direct retinal ganglion cell (RGC) axon growth as well as to assess whether Rho GTPases play a role in translating electrical cues to directional cues.

**Methods:**

Full-thickness, early postnatal mouse retina was cultured in electrotaxis chambers and exposed to EFs of varying strengths (50–200 mV/mm). The direction of RGC axon growth was quantified from time-lapsed videos. The rate of axon growth and responsiveness to changes in EF polarity were also assessed. The effect of toxin B, a broad-spectrum inhibitor of Rho GTPase signaling, and Z62954982, a selective inhibitor of Rac1, on EF-directed growth was determined.

**Results:**

In the absence of an EF, RGC axons demonstrated indiscriminate directional growth from the explant edge. Retinal cultures exposed to an EF of 100 and 200 mV/mm showed markedly asymmetric growth, with 74.2% and 81.2% of axons oriented toward the cathode, respectively (*P* < 0.001). RGC axons responded to acute changes in EF polarity by redirecting their growth toward the “new” cathode. This galvanotropic effect was partially neutralized by toxin B and Rac1 inhibitor Z62954982.

**Conclusions:**

RGC axons exhibit cathode-directed growth in the presence of an EF. This effect is mediated in part by the Rho GTPase signaling cascade.

Restoration of vision in patients blinded by optic neuropathies such as advanced glaucoma requires regeneration of the optic nerve. Cell replacement is a particularly promising approach given recently developed protocols that allow for high-volume production of retinal ganglion cells (RGCs) from human stem cells.^1,2^ Injecting healthy RGCs into the eye, however, is insufficient to regenerate the optic nerve. Although transplanted RGCs have been shown to integrate into the host retina, even displaying some morphologic and electrophysiologic characteristics of mature RGCs,^3,4^ integration rates are low, between 1% and 7%,^4^ and, of the cells that do survive, few sprout axons that extend out of the eye along the optic nerve.^3,5^ These experiments demonstrate that the endogenous cues in the host retina and optic nerve are insufficient to direct the growth of newly transplanted RGCs. Application of an exogenous signal that can direct RGC axon growth out of the eye is necessary for stem cell–based approaches to succeed.

Electrical fields (EFs) have the potential to direct long-distance axon growth.^6–8^ The body has naturally occurring electrical currents,^9,10^ and EFs have been shown to play an important role in directing tissue growth and patterning during normal development.^11^ Several studies have demonstrated that axons of mouse hippocampus, *Xenopus* dorsal root ganglia, and even embryonic chick retinal neurons grow directionally when exposed to an EF in vitro.^12–16^ Currently, there is a phase I clinical trial testing the ability of EFs to direct motor neuron axon growth to restore motor function in patients with spinal cord injury.^6,7^

Drawing on this work, we hypothesized that EFs might exert a similar galvanotropic effect on RGC axon growth. If so, EFs might be exploited to enhance the growth of transplanted RGCs, directing axon growth out of the eye along the optic nerve. Using postnatal mouse retinal explant cultures, we show that RGC axons grow directionally toward the cathode when exposed to an EF. Moreover, we found that directional growth is partially neutralized by toxin B, a broad-spectrum inhibitor of Rho GTPase signaling, as well as by Z62954982, a selective inhibitor of Rac1, suggesting that the Rho GTPase signaling pathway may play a role in translating electrical cues into directional cues.

## Methods

### Retinal Explant Cultures

The use of animals in this study was in accordance with the ARVO Statement for the Use of Animals in Ophthalmic and Vision Research, and was approved by the Ethical Committees at the University of Southern California and the University of California, Davis. CD1 mice were obtained from Charles River Laboratories (Wilmington, MA, USA). Postnatal day (P)0 to P5 CD1 pups were euthanized according to institutional board protocol. Globes were enucleated and placed in ice-cold Dulbecco's modified Eagle's medium (DMEM):F12 media (Gibco, Langley, OK, USA; 11320-082). Retina was isolated and cut into approximately 20 to 50 pieces using a fresh microkeratome. The average area of each retinal explant was 257,124 (±71,328) μm^2^. A plastic transfer pipette was used to transfer the retinal segments into an electrotaxis chamber (see below). Two retinas were plated for each condition. DMEM:F12 medium was removed and tissue culture plates were placed in a 37°C incubator for 15 to 30 minutes to allow the retina to attach to the tissue culture dish. Tissue was then coated with 1000 μL media (see below) and covered with a Linbro plate sealer (Thermo Fisher Scientific, Waltham, MA, USA; ICN7640205) that had been previously cut to a predetermined size and sterilized under UV light for 20 minutes. The tissue culture basin was then filled with media and placed in 37° incubator overnight (12–18 hours).

Tissue culture medium was made with 500 mL Neurobasal A medium (Thermo Fisher Scientific; 10888022), 2.5 mL 100× L-glutamine (Thermo Fisher Scientific, 25030081), 10 mL B27 (Gibco, 17504044), and 5 mL pen/strep. This medium (48 mL) was combined with 5 mL 2.75% methylcellulose in 1× Iscove's modified Dulbecco's medium (IMDM) and 10 mM (4-(2-hydroxyethyl)-1-piperazineethanesulfonic acid) (HEPES). Medium was supplemented with 50 ng/mL brain-derived neurotrophic factor (BNDF) (Peprotech, Rocky Hill, NJ, USA; 450-02), 50 ng/mL ciliary neurotrophic factor (CTNF) (Peprotech, 450-13), and 5 μM forskolin (StemCell Technologies, Cambridge, MA, USA; 72114).

Toxin B (VWR, Radnor, PA, USA; 102946-416) was diluted in Milli-Q purified water (MilliporeSigma, Burlington, MA, USA) and added to medium at time of plating and replenished 1 hour before EF application. Rac1 inhibitor Z62954982 (Sigma, Burlington, MA, USA) was dissolved in dimethyl sulfoxide (DMSO) and added directly to the culture medium 2 hours before EF exposure as previously described.^17^ An equal volume of DMSO was added to control plates.

### Retinal Ganglion Cell Purification

Mouse RGCs were purified using a magnetic-bead separation method that has been previously described.^18^ Briefly, P0 to P5 mouse retinas from CD1 mice were dissected in Mg^2+^/Ca^2+^-free Hanks' balanced salt solution (HBSS). Retinas were then digested for 5 minutes at 37°C in HBSS containing 20 U/mL papain and 0.005% DNase I (Worthington Biochemicals, Lakewood, NJ, USA). Digestion was neutralized with ovomucoid and 0.005% DNase I (Worthington Biochemicals) and then the retina was triturated with a pipette. Dissociated cells were treated with rabbit anti-mouse Thy1.2 antibody conjugated to micrometal beads (130-049-101; Miltenyi Biotech, Auburn, CA, USA) for 15 minutes at room temperature in elution buffer (phosphate-buffered saline with 0.5% bovine serum albumin and 2 mM EDTA; Miltenyi Biotech). RGCs were then purified from the cell suspensions using a metal column in the presence and absence of a magnetic field. Purified RGCs were quantified and then diluted to 0.5 × 10^6^ RGCs/mL in medium (see above). Cells (1 mL) were then plated onto an electrotaxis chamber (see below) and placed in incubator at 37°C overnight for 12 hours.

### Electrotaxis Chamber Preparation

Tissue culture plates were prepared by coating 100-mm plates with 250 μg/mL poly-L-lysine (PLL; Sigma-Aldrich Corp., St. Louis, MO, USA; P1274) overnight or for 6 hours at room temperature. Excess PLL was removed, and plates were dried in the hood for 1.5 hours, then washed three times with PBS. Plates were then coated with 4 μg/mL laminin (Sigma, L2020) overnight. Excess laminin was removed and plates were dried, then washed three times with PBS. Vacuum grease was used to attach cut glass coverslips to build two isolated side-by-side electrotaxis chambers centered around the PLL and laminin coating. Plates were UV sterilized for 20 minutes. Retinal explants or purified RGCs (see above) were then seeded onto the plate. Precut Linbro plate sealer was then placed over the retina and attached to the chamber walls, serving as the roof of the chamber. When fully assembled, the dimensions of the chamber through which current was passed measured 30 × 20 × 0.5 mm.

### Retinal Explant Culture Experiments

EFs were applied as described previously.^19^ Briefly, tissue culture plates were placed in a gas/temperature chamber-controlled inverted Axio Observer 7 microscope (Carl Zeiss, Oberkochen, Germany). Agarose salt bridges were used to connect silver/silver chloride electrodes in beakers of Steinberg's solution to pools of culture medium on either side of the electrotaxis chamber to prevent diffusion of electrode products into the culture medium. Retinal explants were exposed to continuous, direct current with field strengths ranging from 50 to 200 mV/mm at 37°C and 5% CO_2_. EF strengths were measured at the beginning and end of the experiment to ensure consistent EF application. Time-lapsed images were captured with a charge-coupled device camera and the SimplePCI 5.3 imaging system (Hamamatsu Photonics, Hamamatsu City, Japan). Only neurites that demonstrated active growth/elongation or retraction during the time-lapsed videos were quantified. Neurites that showed no change in length during the video were excluded.

### Quantification of Rac1 Activity

Levels of active Rac1-GTP were measured using an absorbance-based G-LISA Rac1 activation assay biochemical kit (BK128; Cytoskeleton, Denver, CO, USA). Briefly, purified RGCs (see above) were cultured overnight in serum-free medium (see above), then exposed to continuous direct current of 200 mV/mm for varying amounts of time. Cell lysates were prepared and GTP-bound Rac1 was measured as directed in the G-LISA protocol. Relative absorption (RA) at 490 nm was determined from experiments performed at least in duplicate.

### Immunofluorescence

After EF exposure, retinal explants were fixed with 4% paraformaldehyde (Polysciences, Philadelphia, PA, USA; 00380-1) for 30 minutes at room temperature and then rinsed twice with PBS. Retinal explants were then permeabilized with 0.3% Triton X-100 (VWR, Radnor, PA, USA) for 15 minutes, blocked with 5% horse serum (VWR) in PBS for 1 hour, and then incubated with mouse anti-MAP2 (1:500; Sigma-Aldrich Corp.; MAB3418) and rabbit anti-Tau (T6402, 1:200; Sigma-Aldrich Corp.) antibodies overnight at 4°C. Explants were rinsed with 0.05% Tween 20 (VWR, 97062-332) four times, then incubated with Alexa Fluor 488–conjugated goat anti-rabbit IgG (1:1000; Jackson ImmunoResearch, West Grove, PA, USA; 111-545-003) and rhodamine-conjugated goat anti-mouse IgG1 (1:1000; Jackson ImmunoResearch 115-295-205) secondary antibodies for 1 hour at room temperature. The explants were then rinsed six times with 0.1% Tween 20, stained with Hoechst for 10 minutes, rinsed twice again, and sealed under 1.5-mm coverslips with Antifade mounting medium (ProLong Gold; Life Technologies, Waltham, MA, USA) before imaging.

### Quantification and Statistics

Direction and speed of axon growth were quantified as previously described.^12^ Briefly, the electrotaxis chamber was aligned so that the EF was parallel to the horizontal axis of the image, with the cathode to the left and the anode to the right of each image. All data were collected from time-lapsed videos. Images were taken at 15- or 20-minute intervals and videoed for a minimum of 4 hours. Images were analyzed using ImageJ (National Institutes of Health, Bethesda, MD, USA) or Zen (Zeiss International, Oberkochen, Germany). Only axons that demonstrated active growth or retraction during the time-lapsed videos were included in the analysis.

**Figure 1:**
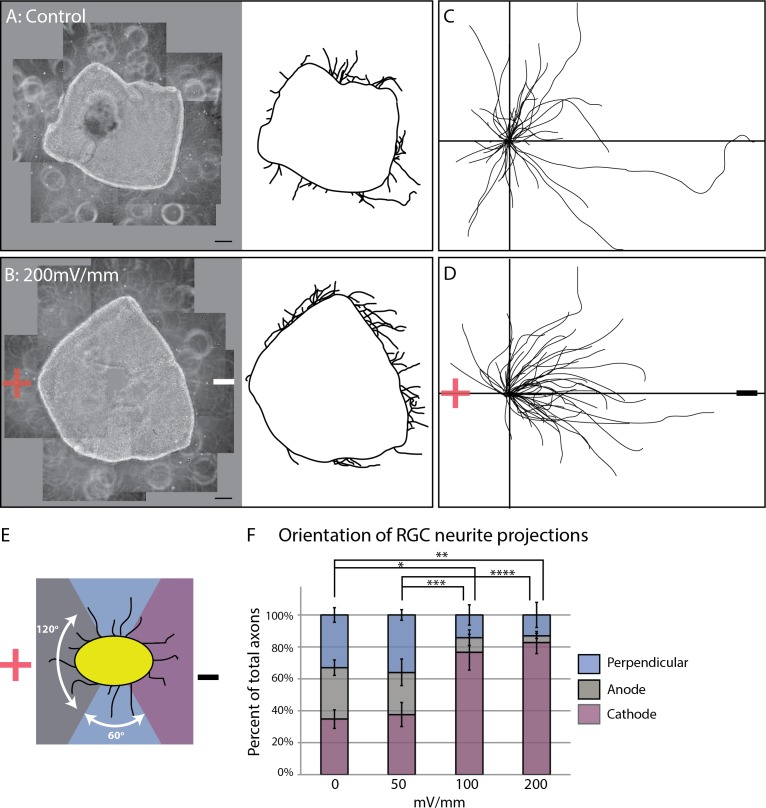
Retinal neurites grow directionally toward the cathode. Retinal explants were grown overnight, then exposed to an EF for 4 hours. (A) Control culture. (B) Culture exposed to EF of 200 mV/mm (cathode on right; anode on left). Left: Composite image of culture. Right: Tracing of explant and all neurites projecting from explant edge. (C, D) Composite of traced neurites from (A, B). See Methods. (E) Schematic of neurite quantification algorithm: gray: anode facing, purple: cathode facing, blue: perpendicular to EF. (F) Retinal explants were exposed to varying strengths of EFs. Neurites projecting from explant edge were quantified as schematized in (E) and represented as percent of total neurites growing toward cathode or anode versus perpendicular to EF. Significantly more neurites demonstrated cathode-directed growth when exposed to an EF of 100 or 200 mV/mm than 0 or 50 mV/mm (*; **; ***; ****P < 0.001; 2-way ANOVA). Error bars represent standard deviation (SD). Scale bar: 100 μm.

An axon was deemed to be growing toward the cathode if its observed growth was within 120° of the negative electrode, measured with the angle function on Zen (Zeiss International; Fig. 1E). Axons that demonstrated growth within 120° of the positive electrode were deemed to be growing toward the anode. The remaining axons were deemed to be growing perpendicular to the EF. Since growing axons extend, retract, and “wobble” by approximately 10°, directionality was assigned as the average angle of growth that was observed during the 4 hours of videography (e.g., if an axon wobbled between 150° and 160°, an angle of 155° was assigned). Axon length was determined by tracing the segment of the axon that was observed to be actively growing in time-lapsed videos: from where the growth cone was first observed at the beginning of the time-lapsed video to its final position at the end of the video. Speed of axon growth was determined by dividing axon length by the total time it took to grow that length (number of frames × time-lapse interval).

For experiments in which EF polarity was switched by 180°, retina was grown as described above, with the cathode located to the left and the anode to the right of the image. After 4 to 6 hours in culture, the polarity was switched by 180° so that the “new” cathode was to the right while the “new” anode was to the left of the image. Axon growth was observed for another 4 to 6 hours. We quantified the angle of growth of all axons that were observed to be actively growing during the first 4 hours (cathode to left; anode to right). The angle of growth was then requantified for these same axons after the EF switch (anode to left; cathode to right). An axon was deemed to be responding to the change in EF polarity if it changed its angle of growth by greater than 10° (either toward or away from the “new” cathode) or if it changed from growing to retracting. Axons that were found to be growing during the first half of the culture and then observed to be retracting during the second half of the experiment were counted and included in the group that lay 180° away (e.g., an axon that was growing toward the left/cathode during the first 4 hours of culture but then was found to be retracting during the second half of the culture period was included in the “new” cathode group, and vice versa). An axon was deemed to be unaffected by the EF switch if its angle of growth changed by less than 10°.

Additional analysis of RGC axonal responses was performed by comparing the average directedness of each culture, as previously described.^20^ Briefly, the angle of axon growth relative to the EF was transformed to a continuous linear variable by taking the cosine of the angle of growth, where 0° was taken to be to the right and 180° to the left side of the image. A cosine θ value closer to 1 would indicate rightward growth while a value closer to −1 indicated leftward growth. Accordingly, a value closer to 0 indicated either stochastic growth or growth perpendicular to the EF. This value was averaged for every culture, allowing comparison of the average directedness between cultures.

In order to determine how rapidly RGC axons respond to changes in EF polarity, the time to when the first change in direction of growth was noticed was tallied for each axon that was seen to be rerouting toward the “new” cathode.

Results are reported from least three independent tissue culture experiments performed with pups from three different litters born from different mating pairs. The percentage of total axons growing toward the cathode versus anode versus perpendicular to the EF, and average axon speed (± standard deviation [SD]) are reported unless otherwise stated. Significant differences were determined using 2-way analysis of variance (ANOVA) followed by Tukey's or Sidak's post hoc test for multiple comparisons using GraphPad Prism 7 (San Diego, CA, USA). *P* < 0.05 was considered to indicate a statistically significant difference. Investigators were blinded to experimental conditions when quantifying the results.

For Figures 1A and 1B, composite pictures of representative control and EF-treated retinal explants were assembled, respectively. From these pictures, Adobe Illustrator (San Jose, CA, USA) was used to hand trace the explant edge as well as all axons observed to be projecting from the edge of the explant. Each axon was traced from the edge of the retinal explant to its growth cone. To demonstrate their overall direction of growth, a composite image of just the axons that were traced was arranged such that the observed origin of each axon (i.e., at the explant edge) originated from a single point (center of crosshairs in Figs. 1C, 1D), then magnified.

Relative levels of Rac1-GTP were calculated by first subtracting the blank, then dividing the average RA of each sample by its paired control (no EF exposure) and multiplying by 100. These values were averaged for each group of experiments and analyzed using 1-way ANOVA with Tukey's multiple comparisons test.

## Results

### Retinal Explant Neurites Grow Directionally Toward the Cathode

Full-thickness, early postnatal mouse retina was cultured in electrotaxis chambers overnight, then exposed to an EF for 4 hours. In the absence of an EF, retinal neurites demonstrated indiscriminate directional growth from the tissue edge. This is depicted in the composite picture and neurite tracings shown in Figures 1A and 1C. Retinal cultures that were exposed to an EF of 200 mV/mm for 4 hours, however, showed marked asymmetry in the direction in which their neurites projected (Figs. 1B, 1D), with most neurites oriented toward the cathode.

We quantified this galvanotropic effect by measuring the angle of neurite growth relative to the EF (see Methods). In control cultures, average directedness of neurite growth was −0.01 (±0.10), indicating either stochastic growth or growth perpendicular to the EF. To discriminate between these two possibilities, we tallied the number of neurites observed to grow within 120° of the cathode versus anode versus perpendicular to the EF (Fig. 1E). As we had no a priori notion as to which direction RGC neurites would grow in, we set our parameter to 120° because this most accurately reflected our three categorical variables: cathode, anode, versus perpendicular growth. In control cultures, neurites demonstrated equal distribution around the explant (Fig. 1F; Supplementary Fig. S1). In contrast, 81.2% of neurites were found to be directed at or turning toward the cathode in cultures exposed to an EF of 200 mV/mm, while only 4.8% and 14.1% were directed toward the anode or perpendicular to the field, respectively (*P* < 0.001; 2-way ANOVA). This is supported by the observation that the average directedness of the 200 mV/mm culture was −0.65 (±0.09; *P* < 0.001; 1-way ANOVA). Explants exposed to 100 mV/mm demonstrated similar asymmetric growth, with an average directedness of −0.46 (±0.10; *P* < 0.001; 1-way ANOVA test). No galvanotropic effect on direction of neurite growth was seen in cultures grown in an EF of 50 mV/mm, whose average directedness was −0.03 (±0.12; *P* = 0.99; 1-way ANOVA).

It is possible that our observation of cathode-directed neurite growth is a secondary consequence of anode-directed axon retraction. We found no difference, however, in the average directedness of retracting neurites between control cultures and those treated with an EF (*P* = 0.71 and 0.99 for 100 and 200 mV/mm, respectively; 1-way ANOVA; Supplementary Fig. S2). Altogether, our results demonstrate that retinal neurites grow directionally toward the cathode when exposed to an EF and that a minimal threshold EF may be necessary to elicit this effect.

### Electrically Responsive Neurites Are RGC Axons

**Figure 2:**
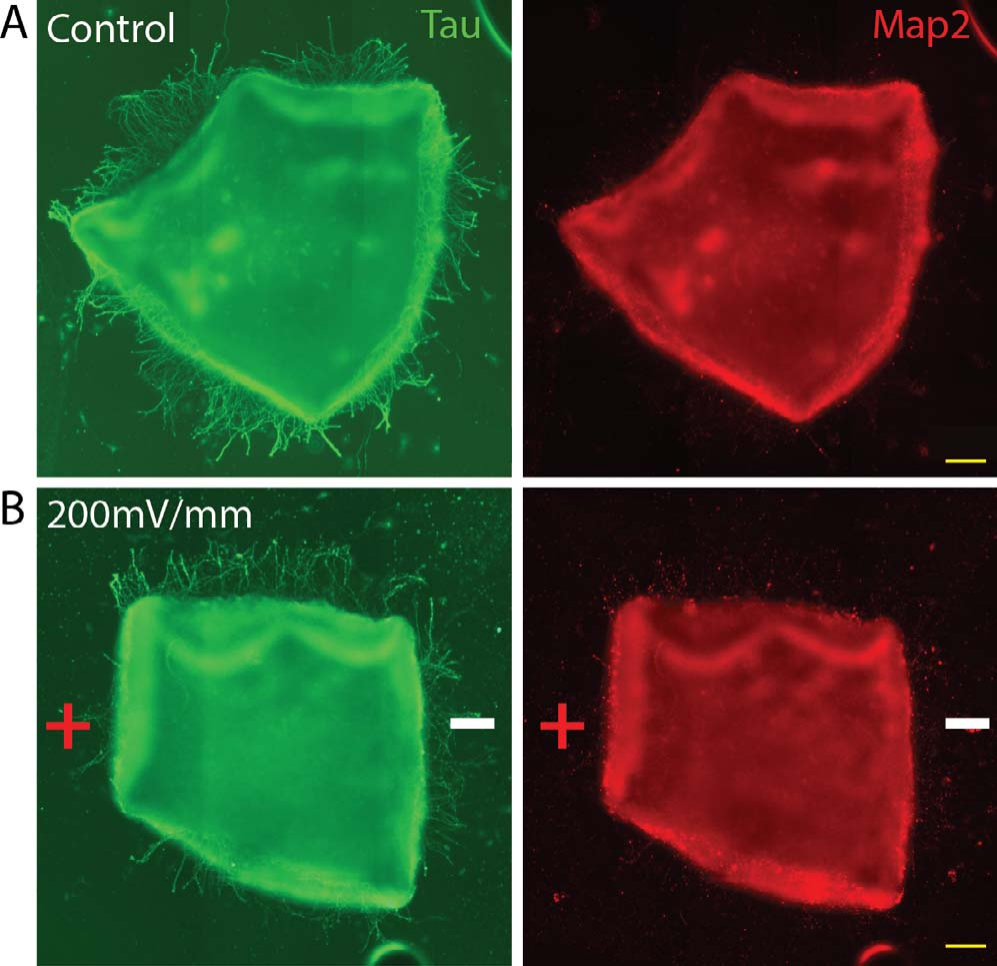
Retinal neurites are axons that demonstrate Tau immunopositivity. Retinal explants were grown for 18 hours, then exposed to an EF of 200 mV/mm for 4 hours. Staining of control (A) and EF-treated (B) explants with anti-Tau and MAP2 antibodies demonstrated that EF-responsive neurites positively stain with axonal (Tau) but not dendritic (MAP2) markers. Scale bar: 100 μm.

Although retinal explant cultures contain a mixed cell population, it is well established that the projections that extend greater than 50 μm from the explant edge represent RGC axons.^21–24^ To further demonstrate that the electroresponsive neurite we quantified as stated above is a RGC axon, we performed immunohistochemistry using anti-MAP2 and anti-Tau antibodies. Anti-MAP2 antibody selectively labels dendrites while axons can be identified by positive labeling with anti-Tau antibody.^25^ Immunostaining of retinal explant cultures exposed to an EF of 200 mV/mm for 4 hours revealed the identity of the EF-responsive neurites to be Tau+/MAP2− axons (Fig. 2). Moreover, the average length of the projections quantified was 130 μm (±22 SD), further suggesting that EF-responsive neurites are RGC axons.

### Electrical Field Does Not Affect the Rate of RGC Axon Growth

**Figure 3:**
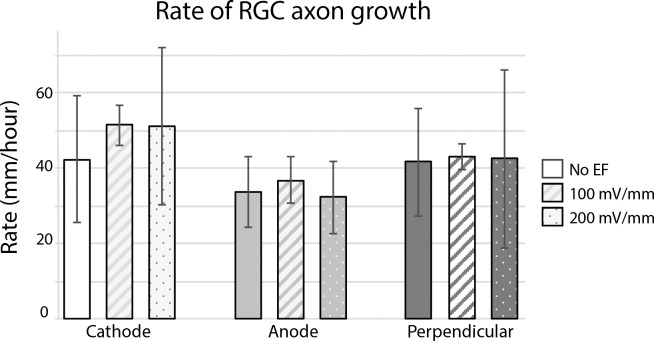
EF does not affect rate of RGC axon growth. Retinal explant cultures were grown for 18 hours, then exposed to an EF of 100 or 200 mV/mm for 5 hours. Rate of RGC axon growth was assessed by measuring maximum length of observed growth and time to maximal length (see Methods). No difference in rate of growth was noted in EF-treated axons compared to control cultures. Error bars represent SD.

To further characterize the effect of EFs on RGC axon growth, we quantified the rate of RGC axon growth by measuring the time required for each axon to reach its maximal length. An average rate of 42.3 μm/hour (±16.8 SD) growth was measured in control cultures, similar in magnitude to that reported for *Xenopus* spinal neurons.^12^ EF exposure appeared to increase the rate of growth of cathode-directed axons (Fig. 3; Supplementary Fig. S3), although this effect was not found to be statistically significant (*P* = 0.64 and 0.60 for 100 and 200 mV/mm, respectively, 2-way ANOVA). No difference was detected in anodally or perpendicularly directed axons exposed to 100 or 200 mV/mm (Fig. 3; Supplementary Fig. S3).

### RGC Axons Respond Acutely to Changes in EF Polarity

**Figure 4:**
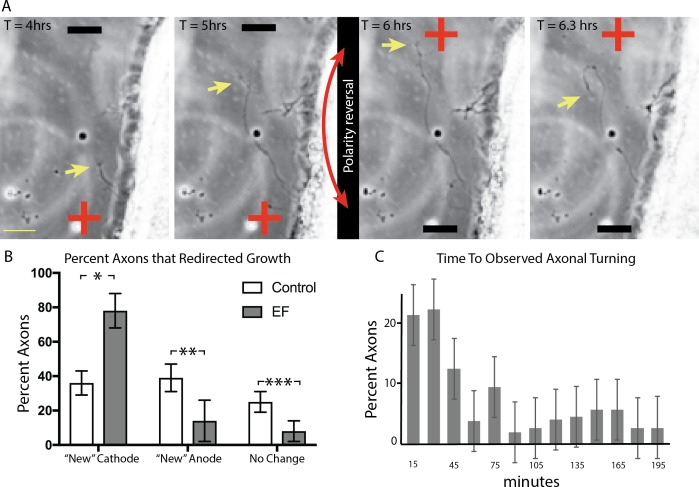
RGC axons change direction of growth in response to reversal in EF polarity. (A) Retinal explants were grown overnight, then exposed to an EF of 100 mV/mm for 6 hours, after which polarity was reversed by 180°. Explants were then monitored for 6 more hours. An axon can be seen reversing its direction of growth within a single time frame (20 minutes) of switching EF polarity. Also see Supplementary Video S1. (Yellow arrow points to representative growth cone.) (B) Explants were grown overnight, then exposed to an EF of 200 mV/mm for 4 hours. The polarity of the EF was then reversed by 180° and cultures were observed for another 4 hours. The percent of axons seen redirecting their growth after the EF switch toward the “new” cathode, “new” anode, versus no reaction was quantified (see Methods). Results demonstrate that significantly more axons redirected their growth toward the “new” cathode than random turns seen in culture. (*P < 0.001; **; ***P < 0.05; 2-way ANOVA). (C) Of the axons that redirected their growth toward the “new” cathode in (B), the number of time frames that had elapsed since the EF switch and this change in direction of growth could be observed was tallied and recorded. Error bars represent SD. Yellow scale bar: 25 μm.

We wondered whether exposure to an EF commits the RGC axon to grow along its initial trajectory or if RGC axons retain the ability to respond to acute changes in EF polarity by changing the direction of their growth. To test this, we exposed retinal explant cultures to an EF for 4 hours and then acutely reversed the polarity of the EF by 180° and monitored their growth for another 4 hours (Fig. 4; Supplementary Video S1). Analysis of time-lapsed microscopy videos demonstrated that an average of 78% of axons redirected their growth toward the “new” cathode and 14% toward the “new” anode, while 8% did not react to the switch in EF polarity (continued to grow along the initial course). This turning was observed more frequently than the random turns seen in control cultures (36% turned to the right, 39% turned to the left, 25% made no turns when comparing orientation of growth during the first 4 hours to the second 4 hours of the culture period; *P* < 0.001, 2-way ANOVA; Fig. 4B; Supplementary Figs. S4A, S4B).

This finding was supported by analysis of the average directedness of axon growth in each condition. In cultures treated with an EF of 200 mV/mm, the average direction of growth was −0.47 (±0.15), indicating average leftward growth toward the cathode, and changed to ±0.60 (±0.22) after the EF was switched by 180°, indicating an overall rightward shift toward the “new” cathode. This was significantly different from control cultures where the average directedness of growth was ±0.03 (±0.23) during the first 4 hours of culture, indicating neither leftward nor rightward growth, and changed minimally to ±0.08 (±0.10) in the latter 4 hours of culture (Supplementary Fig. S4C; *P* < 0.001; 2-way ANOVA). Altogether, these data suggest that RGC axons respond to changes in EF polarity by changing their growth and do so by redirecting their growth toward the “new” cathode.

To understand the acuity with which cathode-directed RGC axons can sense and respond to changes in EF polarity, we quantified the number of time frames that elapsed from when the EF polarity was switched and a change in the direction of axon growth could be detected. Of the axons that rerouted their growth toward the “new” cathode, on average, 21% were observed to have done so within the first frame (15 minutes) and 55% were observed to have done so within three frames (45 minutes) (Fig. 4C; Supplementary Fig. S4E).

**Figure 5:**
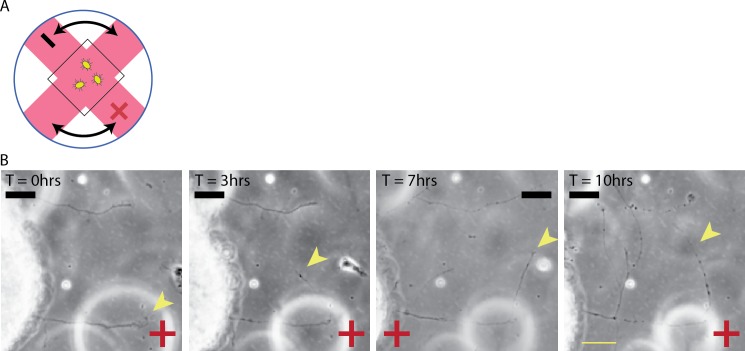
RGC axons shift direction of growth in response to acute changes in EF angle. (A) Schematic of electrotaxis chamber that allowed EF polarity to be changed by 90°. (B) Explants were grown for 12 hours, then exposed to an EF of 200 mV/mm (T = 0). Cathode-directed growth can be seen at T = 3 hours. At T = 4 hours, the direction of the EF was shifted 90° clockwise and the RGC axon can be seen shifting toward the “new” cathode at T = 7 hours. At T = 7 hours, the cathode was shifted 90° counterclockwise, and at T = 10 hours, the RGC axon again shifted its growth toward the “new” cathode. See Supplementary Video S2. (Yellow arrowhead points to representative growth cone.) Yellow scale bar: 50 μm.

An acute response to change in EF polarity was also observed with smaller changes in field angle. We designed a tissue culture chamber where the angle of the EF could be acutely changed by 90° (Fig. 5A) as opposed to the 180° change presented in Figure 4. As can be seen in Figure 5B and Supplementary Video S2, retinal axons acutely respond to multiple changes in EF polarity by making sharp 90° turns. These experiments demonstrate that even after exposure to an EF for multiple hours, RGC axons retain the ability to detect and rapidly respond to changes in EF polarity.

### EF-Induced Directional Growth Is Partially Neutralized by Broad-Spectrum Rho GTPase Inhibitor Toxin B

The underlying mechanisms through which EFs direct axon growth are unknown and under active investigation by our group and others.^11,26^ One mechanism through which EFs have been proposed to direct neurite growth is by inducing asymmetric localization or asymmetric activation of cell surface receptors and channels, including Ca^2+^ channels. As downstream effectors of Ca^2+^ signaling, the Rho family of GTPases (Rho, Rac, and Cdc42) are proposed to control neurite growth by interfacing with components of the cytoskeleton, with RhoA inducing growth cone collapse and neurite retraction and Rac1/Cdc42 promoting neurite extension.^27^ Local activation of RhoA signaling by EFs on anode-facing projections may lead to retraction of anode-facing neurites, thereby providing a permissive environment for growth of cathode-oriented neurites. Conversely, local activation of Rac1/Cdc42 on cathode-facing projections may lead to neurite extension on cathode-facing neurites. This is the mechanism by which EFs have been proposed to mediate control of neurite growth of *Xenopus* spinal neurons.^12^

**Figure 6:**
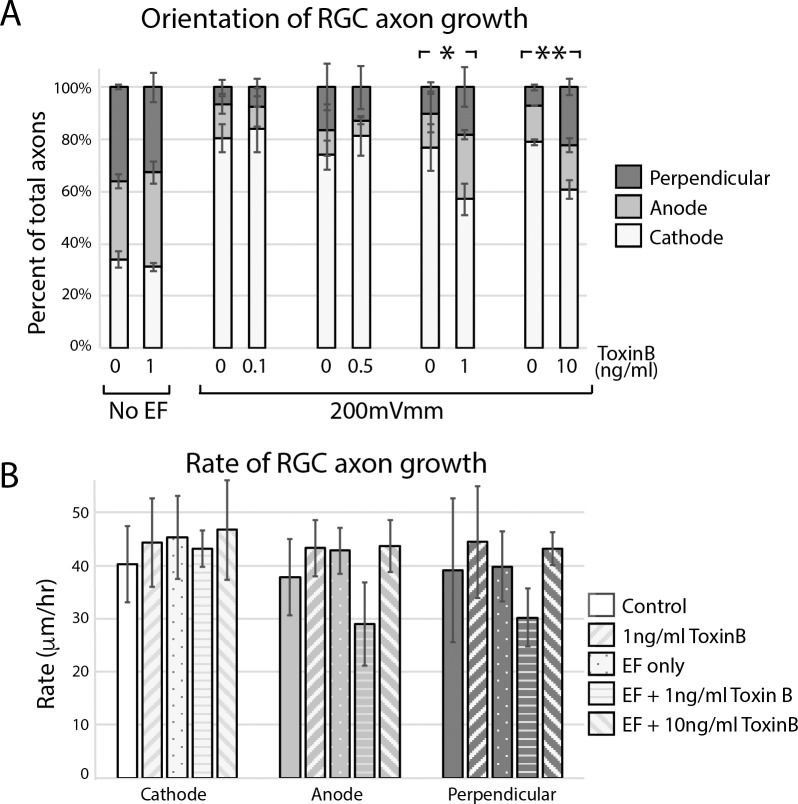
Toxin B partially neutralizes cathode-directed growth of RGC axons. Explants were grown for 12 hours in varying concentrations of toxin B, then exposed to an EF of 200 mV/mm. Toxin B was replenished 1 hour before initiating EF exposure. (A) Significantly fewer axons were observed to grow toward the cathode in explants exposed to 1 ng/mL (*P < 0.001; 2-way ANOVA with Tukey's multiple comparison test) and 10 ng/mL (**P < 0.05; 2-way ANOVA with Tukey's multiple comparison test). No effect was noted when 0.1 or 0.5 ng/mL toxin B was added to culture. Error bars represent SD. (B) The effect of 1 and 10 ng/mL toxin B on rate of axon growth, with and without an EF, was quantified. Toxin B had no significant effect on rate of axon growth. Error bars represent SD.

To test whether Rho GTPase signaling plays a role in mediating EF-induced control of RGC axon growth, we treated retinal explant cultures with a broad-spectrum inhibitor of the Rho GTPase signaling pathway, toxin B.^28^ In explants exposed to 1 ng/mL toxin B, significantly fewer axons demonstrated cathode-directed growth (Fig. 6A; *P* < 0.001; 2-way ANOVA). Similar results were seen with 10 ng/mL toxin B (Fig. 6A; Supplementary Fig. S5). Of note, no effect was detected in cultures treated with 0.1 or 0.5 ng/mL toxin B (Fig. 6A; Supplementary Fig. S5). We also quantified the effect of toxin B alone on rate of RGC axon growth. As seen in Figure 6B and Supplementary Figure S6, toxin B did not significantly affect the rate of RGC axon growth, even in the absence of an EF.

### Rac1-Specific Inhibition Neutralizes Cathode-Directed Growth of RGC Axons

**Figure 7:**
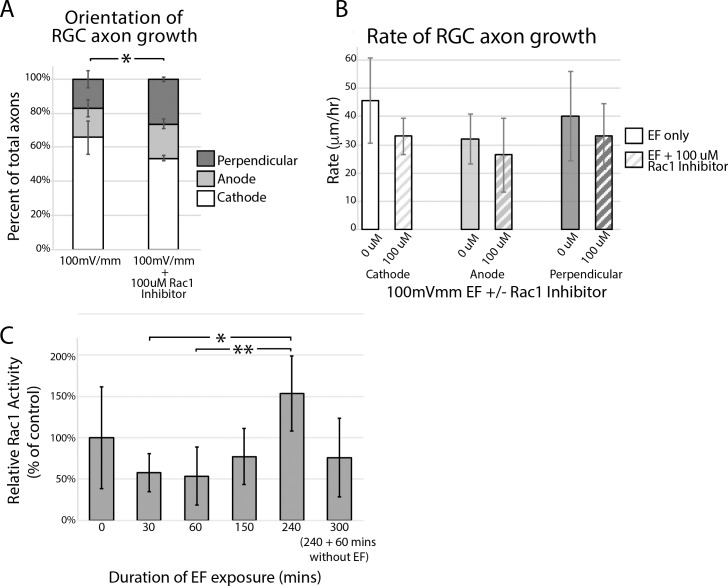
Rac1 inhibitor Z62954982 neutralizes cathode-directed growth. Retinal explant cultures were grown for 12 hours, then treated with 100 μM Z62954982, a selective small molecule inhibitor of Rac1. Two hours later, explants were exposed to an EF of 100 mV/mm for 4 hours. (A) Percent axons growing toward the cathode versus anode versus perpendicular to the EF was quantified. Significantly fewer axons were found to be growing toward the cathode in Z62954982-treated cultures compared to EF controls (*P < 0.05; 2-way ANOVA with Sidak's multiple comparison test). (B) 100 μM Z62954982 appeared to decrease the rate of growth of cathode-directed axons, although this effect was not found to be statistically significant (P > 0.05; n = 3, 2-way ANOVA with Sidak's multiple comparison test). (C) Cultures of purified RGCs were grown overnight and then exposed to an EF of 200 mV/mm for varying durations of time. Level of Rac1-GTP relative to control cultures was quantified from cell lysates. Compared to baseline, experiments demonstrated a trend toward decreased levels of Rac1-GTP 30 minutes (P = 0.43, n = 7; 1-way ANOVA with Tukey's multiple comparison test) and 60 minutes (P = 0.44, n = 6; 1-way ANOVA with Tukey's multiple comparison test) after EF exposure, although this was not found to be statistically significant. Relative to 30- and 60-minute treatment times, Rac1-GTP levels significantly increased after 4 hours of EF exposure (*;**P < 0.05; n = 5; 1-way ANOVA with Tukey's multiple comparison test), then trended toward decreasing after the EF was removed (P = 0.08 for 300 minutes relative to 240 minutes; n = 5; 1-way ANOVA with Tukey's multiple comparison test).

As toxin B is a broad-spectrum inhibitor of the Rho GTPase signaling pathway, we sought to more directly test the role of the Rho kinase cascade in translating EFs into directional cues by culturing retinal explants with a previously validated, selective small molecule inhibitor of Rac1, Z62954982. Z62954982 readily diffuses across cell membranes and has been shown to disrupt the Rac1/Tiam1 complex, thereby decreasing cytoplasmic levels of active Rac1 (GTP-bound Rac1), without affecting the activity of other Rho GTPases, namely, Cdc42 or RhoA.^29^ Compared to cultures exposed to EF alone, 100 μM Z62954982 markedly neutralized cathode-directed growth of RGC axons (Fig. 7A; Supplementary Fig. S7; *P* < 0.05; 2-way ANOVA with Sidak's multiple comparison test). Unlike toxin B, Z62954982 appeared to decrease the rate of growth in cathode-directed axons (Fig. 7B), although this effect was not statistically significant (*P* = 0.15; 2-way ANOVA with Sidak's multiple comparisons test). These data implicate a likely role for the Rho GTPase pathway in responding to EF exposure.

### EF Exposure Leads to Increased Levels of Rac1-GTP

To test whether EF exposure leads to increased concentration of activated Rac1, purified RGCs were cultured overnight, then exposed to an EF of 200 mV/mm for varying lengths of time. RGCs were cultured at a low density of 500,000 cells/1000 mm^3^ so as to minimize the effect cell–cell interaction may have on Rac1-GTP levels. Cytoplasmic levels of GTP-bound Rac1 were quantified using an ELISA-based kit. Our experiments suggest a trend toward decreased levels of Rac1-GTP with short-term (30 and 60 minutes) EF exposure (Fig. 7C; *P* = 0.43 and *P* = 0.44, respectively, *n* = 6; 1-way ANOVA with Tukey's multiple comparison test). Compared to 30 and 60 minutes, 4 hours of exposure to an EF of 200 mV/mm was associated with a 1.63-fold increase in levels of activated Rac1 (Fig. 7C; *P* < 0.05 for both 30 and 60 minutes; *n* = 5; 1-way ANOVA with Tukey's multiple comparison test). Relative levels of Rac1-GTP appeared to decline to baseline after EF exposure was removed (Fig. 7C; *P* = 0.08; *n* = 5; 1-way ANOVA with Tukey's multiple comparison test). Altogether, these data suggest that the Rho GTPase signaling pathway, in part, plays a role in translating EFs into directional cues in RGCs.

## Discussion

Although cell transplantation–based approaches are a promising strategy for optic nerve regeneration, these approaches still face daunting challenges before visual function can be restored. One of the main problems is that transplanted RGCs do not readily extend an axon out of the eye. Whether stunted axonogenesis results from an absence of directional cues and/or failure to overcome inhibitory signals is unknown.^30^ There is much interest in the potential of EFs to direct long-distance axon growth. Here, we have shown that postnatal mouse RGC axons grow toward the cathode in the presence of an EF (Fig. 1). Directed growth was not simply a result of EF-induced neurite retraction as we detected no difference in the direction in which axons retracted in EF-treated versus control cultures (Supplementary Fig. S2), albeit few retracting axons were noted overall in our cultures. Our findings expand upon previously published work with embryonic chick retina showing that retinal axons grow toward the cathode when cultured in the presence of an EF.^31^

As the neuritic potential of the central retina is greater than that of the peripheral retina, the argument could be made that the orientation of our retinal explants relative to the EF could account for our findings. Indeed, previous groups have exploited retinal asymmetry for experimental purposes.^31^ We believe this to be an unlikely explanation for our findings as our experiments were performed with retinal tissue cut into much smaller pieces than the experiments referenced above (see Methods). In our experiments, each condition consisted of two retinas cut into 20 to 50 pieces each. As these pieces were allowed to settle in the electrotaxis chamber at random, each condition had the same probability of peripheral versus central retina being oriented parallel or perpendicular to the EF. Additionally, we showed that RGC axons reroute their direction of growth in reaction to a reversal in EF polarity (Fig. 4), with most redirecting toward the “new” cathode, and are able to sense small angle changes in EF polarity (Fig. 5). Our ability to detect a response to the change in EF polarity argues that the EF is directly influencing the direction of RGC axon growth rather than the orientation of the retinal tissue relative to the EF, which was not changed during these experiments.

Interestingly, we did not find EF exposure to affect the rate of RGC axon growth. There is significant variability in the literature regarding the effect of EF exposure on speed of axon growth. While physiologic EFs were found to increase the rate of axon extension toward the cathode^12,15^ and decrease the rate of growth toward the anode in *Xenopus* spinal neurons,^12^ no effect on growth rate was noted with rat motor neurons.^32^ Different experimental conditions likely underlie the differences between our findings and those reported in the literature.

The underlying mechanisms through which cells translate EFs into directional cues are not well understood but are being actively investigated.^26,33^ It is believed that EFs lead to redistribution of cell membrane proteins, allowing for local activation of newly clustered ionic channels, and/or induce asymmetric activation of voltage-sensitive ion channels, such as voltage-gated Ca^2+^ channels. Support for this comes from experiments that have shown Ca^2+^ influx into keratinocytes after EF exposure^34^ and shown that galvanotaxis is neutralized with concavalin A,^35^ Ca^2+^ channel blockers, and when Ca^2+^ is removed from the tissue culture media.^36^ This led us and others^12^ to suspect a role for the Rho family of GTPases, known downstream effectors of Ca^2+^ signaling, in mediating EF signals. Indeed, we show that toxin B, a broad-spectrum inhibitor of Rho GTPase signaling, partially neutralized cathode-directed RGC axon growth without affecting the rate of axon growth (Fig. 6). Further support for this hypothesis can be drawn from experiments showing (1) that selective inhibition of Rac1 neutralized cathode-directed RGC axon growth (Fig. 7A) and (2) that EF application is associated with a rise in levels of activated Rac1 in cultures of purified RGCs (Fig. 7C).

Interestingly, Rac1 inhibition appeared to have a more profound effect on neutralizing EF-mediated RGC axon galvanotaxis than toxin B (Fig. 7A compared to Fig. 6A). Similar findings were reported in *Xenopus* spinal neurons.^12^ A possible explanation for this finding is that Rac1 is a known promoter of neurite extension while toxin B is a ubiquitous inhibitor of Rho GTPases that include promotors of neurite extension (e.g., Rac1 and Cdc42) as well as promotors of neurite retraction (e.g., RhoA). It is possible that asymmetric inhibition of RhoA relative to Rac1/Cdc42, or even other unknown signaling molecules, by toxin B accounts for the more muted effect on electrotaxis than was seen with the selective Rac1 inhibitor, Z62954982. This asymmetry could be dose dependent and explain why 1 ng/mL was more effective at neutralizing cathode-directed growth than 10 ng/mL (Fig. 6A). Some support for this hypothesis can be found in experiments that quantified the effect of toxin B and Z62954982 on the rate of RGC axon growth. Toxin B had no effect (Fig. 6B) on rate of axon growth while Z62954982 appeared to decrease the growth rate of cathode-directed axons (Fig. 7B), although this effect was not statistically significant. Opposing effects between toxin B and Rac1 inhibitors on rate of axon growth have been reported by others^12^ and could be consistent with selective inhibition of a growth promotor compared to simultaneous inhibition of growth promotors and retractors.

While analyzing RGC axonal responses to change in EF polarity, we were able to document that 21% of cathode-responsive axons responded to the EF switch within 15 minutes and that over 50% responded within 45 minutes (Fig. 4). Our ability to accurately assess this change was limited by the fact that we set our imaging interval to 15 minutes. Nevertheless, this short time frame suggests that EFs may be acting directly on RGCs as opposed to directing axon growth through another cell type in our culture system. Ultimate proof of this will require characterizing the response of purified RGCs to an EF.

Although we were able to detect a significant response to the EF within 15 minutes of changing polarity (Fig. 4), the finding that 30 minutes of EF exposure led to decreased levels of activated Rac1 and that the rise in Rac1-GTP levels did not occur until after 4 hours of EF exposure (Fig. 7C) suggests that other, Rac1-independent pathways, are also at play. Future studies are needed to determine what role other Rho GTPase effectors (e.g., RhoA, Cdc42) play in mediating cellular responses to EFs.

Understanding the mechanisms underlying EF-directed axonal growth holds great potential to advance the field of optic nerve regeneration. Our work suggests that exogenous application of EFs to the optic nerve may be a useful adjunct to cell transplantation–based techniques for optic nerve regeneration. It will ultimately be necessary to demonstrate that (1) EFs can be safely applied to the optic nerve and that (2) EFs direct RGC axon growth in vivo. To have a significant impact, EFs would need to override endogenous anti-axonogenesis signals (e.g., myelin, inflammatory molecules, and gliotic scar).^30^ Promising support for the utility of EFs can be found in recent in vivo studies showing that application of an EF along the rostral migratory stream of adult rat brains was able to reroute migrating neural stem cells.^37^ Instead of migrating rostrally from the subventricular zone (SVZ) to the olfactory bulb, labeled stem cells were found to migrate caudally back toward the SVZ in the presence of an EF, a phenomenon that is never seen in situ. What these experiments show is that EFs can not only direct cell migration in vivo but can also override endogenous directional cues and reroute cells to novel targets. Additional support comes from experiments in which EFs were continuously applied to transected guinea pig spinal cords for 1 month: Significantly more animals had axons that extended up to, around, and even through the transection site than control animals.^38^

Studies suggest that simply promoting axon growth does not ensure proper axonal targeting: In cases where long-distance RGC axon growth was achieved in vivo, many axons were observed to overshoot their target^3^ or grow toward aberrant targets (e.g., toward the other eye or back on themselves).^39^ This implies a need not just for signals that promote axon growth but also ones that direct axon growth. Promising results were seen when neural activation was combined with increases in proregenerative signals (mammalian target of rapamycin, mTOR): Target-specific, long-distance RGC axon growth was seen with partial regain of function.^40^ What role EFs may have in further bridging this gap remains to be tested. Ultimately successful optic nerve regeneration will require combinatorial approaches that exploit molecular signals to support intrinsic axon growth and provide exogenous signals such as EFs to provide directional cues.

## Supplementary Material

Supplement 1

Supplement 2

Supplement 3

Supplement 4

Supplement 5

Supplement 6

Supplement 7

Supplement 8

Supplement 9

Supplement 10
