# Supplementary material for: Physiologic Electrical Fields Direct Retinal Ganglion Cell Axon Growth In Vitro
Source: Invest Ophthalmol Vis Sci. 2019 Aug;60(10):3659–68. doi: 10.1167/iovs.18-25118 (PMC6716951; doi:10.1167/iovs.18-25118)
Supplement: Supplement 3 [file iovs-60-10-10_s03.pdf]

Supplemental Figure 1

A

	Control	50mV/mm	100mV/mm	200mV/mm
n experiments	10	3	3	4
n axons	1234	871	326	303
Cathode	33.4% (± 4.3%)	37.7% (± 7.6%)	74.2% (± 7.6%)	81.2% (± 9.3%)
Anode	32.0% (± 5.0%)	26.4% (± 8.3%)	9.0% (± 4.9%)	4.8% (± 2.4%)
Perpendicular	34.6% (± 3.9%)	35.9% (± 3.4%)	16.8% (± 3.2%)	14.1% (± 10.0%)

B

	**Cathode**				
**Cathode**		Control	50mV/mm	100mV/mm	200mV/mm
	Control	N/A			
	50mV/mm	0.6646	N/A		
	100mV/mm	**<0.0001**	**<0.0001**	N/A	
	200mV/mm	**<0.0001**	**<0.0001**	0.3856	N/A

	**Anode**				
**Anode**		Control	50mV/mm	100mV/mm	200mV/mm
	Control	N/A			
	50mV/mm	0.4516	N/A		
	100mV/mm	**<0.0001**	**0.0027**	N/A	
	200mV/mm	**<0.0001**	**<0.0001**	0.7710	N/A

	**Perpendicular**				
**Perpendicular**		Control	50mV/mm	100mV/mm	200mV/mm
	Control	N/A			
	50mV/mm	0.9856	N/A		
	100mV/mm	**0.0001**	**0.0009**	N/A	
	200mV/mm	**<0.0001**	**<0.0001**	0.9255	N/A

Figure S1: Retinal neurites grow towards the cathode in the presence of an electrical field. Retinal explant cultures were grown for 18 hours, then exposed to an EF for 4 hours. (A) Percent of total axons projecting towards the cathode, anode, or perpendicular to the EF (see METHODS). Values represent mean with SD. Number of experiments and total axons quantified are listed per condition. (B) P-values from two-way analysis of variance of data of (A) with Tukey's multiple comparisons test.
