# Supplementary material for: Physiologic Electrical Fields Direct Retinal Ganglion Cell Axon Growth In Vitro
Source: Invest Ophthalmol Vis Sci. 2019 Aug;60(10):3659–68. doi: 10.1167/iovs.18-25118 (PMC6716951; doi:10.1167/iovs.18-25118)
Supplement: Supplement 4 [file iovs-60-10-10_s04.pdf]

Supplemental Figure 2

A

	Control	50mV/mm	100mV/mm	200mV/mm
n experiments	9	3	3	3
n axons	66	67	48	131
Directedness	-0.34 (± 0.47)	-0.16 (± 0.60)	0.03 (± 0.59)	-0.28 (± 0.62)

B

	Control	50mV/mm	100mV/mm	200mV/mm
Control	N/A			
50mV/mm	0.9517	N/A		
100mV/mm	0.7099	0.9687	N/A	
200mV/mm	0.9981	0.9914	0.8808	N/A

Figure S2: Retinal neurites exhibit cathode-directed growth. (A) Average directedness of retinal neurites was calculated from cultures described in Figure 1. Number of experiments and total axons quantified are listed per condition. Values represent mean with SD. (B) P-values from one-way analysis of variance of data of (A) with Tukey's multiple comparisons test.
