# Supplementary material for: Physiologic Electrical Fields Direct Retinal Ganglion Cell Axon Growth In Vitro
Source: Invest Ophthalmol Vis Sci. 2019 Aug;60(10):3659–68. doi: 10.1167/iovs.18-25118 (PMC6716951; doi:10.1167/iovs.18-25118)
Supplement: Supplement 5 [file iovs-60-10-10_s05.pdf]

Supplemental Figure 3

A

	Control	100mV/mm	200mV/mm
n experiments	7	3	4
n axons	630	321	277
Cathode	42.3 (± 16.8)	51.4 (± 5.2)	51.1 (± 20.8)
Anode	33.9 (± 9.3)	36.8 (± 6.2)	32.3 (± 9.5)
Perpendicular	41.6 (± 14.2)	43.0 (± 3.4)	42.6 (± 23.5)

B

**Cathode**	Control	100mV/mm	200mV/mm
Control	N/A		
100mV/mm	0.6358	N/A	
200mV/mm	0.5958	0.9997	N/A

**Anode**	Control	100mV/mm	200mV/mm
Control	N/A		
100mV/mm	0.9516	N/A	
200mV/mm	0.9845	0.9120	N/A

**Perpendicular**	Control	100mV/mm	200mV/mm
Control	N/A		
100mV/mm	0.9883	N/A	
200mV/mm	0.9936	0.9989	N/A

Figure S3: EF does not affect rate of RGC axon growth. (A) Retinal explant cultures were grown for 18 hours, then exposed to an EF of 100 or 200 mV/mm for 5 hours. Rate of RGC axon growth was assessed by measuring maximum length of observed growth and time to maximal length (see METHODS). No difference in rate of growth was noted in EF-treated axons compared to control cultures. Number of experiments and total axons quantified are listed per condition. Error bars represent SD. (B) P-values from two-way analysis of variance of data from (A) with Tukey's multiple comparisons test.
