# Supplementary material for: Physiologic Electrical Fields Direct Retinal Ganglion Cell Axon Growth In Vitro
Source: Invest Ophthalmol Vis Sci. 2019 Aug;60(10):3659–68. doi: 10.1167/iovs.18-25118 (PMC6716951; doi:10.1167/iovs.18-25118)
Supplement: Supplement 6 [file iovs-60-10-10_s06.pdf]

Supplemental Figure 4

A

Percent total axons	Changed towards "new" cathode	Changed towards "new" anode	No reaction to change in EF polarity	n experiment	n axons
Control	36% (± 7%)	39% (± 8%)	25% (± 6%)	4	271
200 mV/mm	78% (± 10%)	14% (± 12%)	8% (± 6%)	4	162

B

Two-way ANOVA	Changed towards "new" cathode	Changed towards "new" anode	No reaction to change in EF polarity
Control vs 200 mV/mm	**<0.0001**	**0.0017**	**0.0320**

C

Average Directedness	Culture 0-4 hours; Cathode towards left	Culture 4-8 hours; Cathode towards right	Net change in axon directedness
Control	0.03 ± 0.23	0.08 ± 0.10	0.05 ± 0.13
200 mV/mm	-0.47 ± 0.15	+0.60 ± 0.22	1.07 ± 0.31

D

Two-way ANOVA	Control Culture T=0-4 hrs	Control Culture T=4-8 hrs	EF Culture Cathode to left	EF Culture Cathode to right
Control Culture T=0-4 hrs	N/A	0.9994	**0.0134**	**0.0051**
Control Culture T=4-8 hrs		N/A	**0.0067**	**0.0101**
EF Culture Cathode to left			N/A	**<0.0001**
EF Culture Cathode to right				N/A

E

Number of minutes elapsed after switch in EF polarity	Average percent total axons observed switching (± standard deviation)
15	21.27 (± 11.05)
30	22.14 (± 17.01)
45	12.39 (± 6.04)
60	3.91 (± 5.25)
75	9.54 (± 8.62)
90	2.06 (± 2.40)
105	2.77 (± 5.55)
120	4.12 (± 4.81)
135	4.54 (± 9.09)
150	5.76 (± 4.69)
165	5.83 (± 4.72)
180	2.77 (± 5.55)
195	2.85 (± 3.67)

Figure S4: RGC axons change direction of growth in response to reversal in EF polarity. Explants were grown overnight for 12 hours, then exposed to an EF of 200 mV/mm for 4 hours. The polarity of the EF was then reversed by 180 degrees and cultures were observed for another 4 hours. The percent of axons seen re-directing their growth after the EF switch towards the "new" cathode, "new" anode, vs no reaction to was quantified and shown in (A), see METHODS. Number of experiments and total axons quantified are listed per condition. Values represent mean with SD. (B) P-values from two-way analysis of variance of data from (A). (C) Measurement of average culture directedness (see METHODS) shows increased rightward shift (towards "new" cathode) in EF treated cultures compared to control cultures. (D) P-values from two-way analysis of variance of experiment described in (C). (E) Of the axons that redirected their growth towards the "new" cathode in (B), the number of time elapsed since the EF switch for this change in direction to be observed was tallied and recorded. Error represents SD.
