# Supplementary material for: Physiologic Electrical Fields Direct Retinal Ganglion Cell Axon Growth In Vitro
Source: Invest Ophthalmol Vis Sci. 2019 Aug;60(10):3659–68. doi: 10.1167/iovs.18-25118 (PMC6716951; doi:10.1167/iovs.18-25118)
Supplement: Supplement 7 [file iovs-60-10-10_s07.pdf]

Supplemental Figure 5

A

% total axons	200mV/mm + ToxinB				
	0 ng/ml	0.1 ng/ml	0.5 ng/ml	1 ng/ml	10 ng/ml
Cathode	78% (± 6%)	84% (± 9%)	81% (± 8%)	57% (± 6%)	61% (± 4%)
Anode	12% (± 6%)	8% (± 7%)	6% (± 1%)	25% (± 2%)	17% (± 3%)
Perpendicular	10% (± 6%)	8% (± 3%)	13% (± 8%)	18% (± 8%)	22% (± 3%)
Directedness	-0.61 (± 0.06)	-0.66 (± 0.01)	-0.67 (± 0.08)	-0.31 (± 0.04)	-0.37 (± 0.07)
n experiment	13	3	3	4	3
n axons	849	154	110	478	224

B

% total axons			**Cathode**				
			200mV/mm + ToxinB				
			0 ng/ml	0.1 ng/ml	0.5 ng/ml	1 ng/ml	10 ng/ml
**Cathode**	200mV/mm + ToxinB	0 ng/ml	N/A				
		0.1 ng/ml	0.9567	N/A			
		0.5 ng/ml	>0.9999	>0.9999	N/A		
		1 ng/ml	**<0.0001**	**<0.0001**	**<0.0001**	N/A	
		10 ng/ml	**0.0027**	**0.0011**	**0.0084**	0.9999	N/A

% total axons			**Anode**				
			200mV/mm + ToxinB				
			0 ng/ml	0.1 ng/ml	0.5 ng/ml	1 ng/ml	10 ng/ml
**Anode**	200mV/mm + ToxinB	0 ng/ml	N/A				
		0.1 ng/ml	0.9991	N/A			
		0.5 ng/ml	0.9567	>0.9999	N/A		
		1 ng/ml	**0.0209**	**0.0264**	**0.0068**	N/A	
		10 ng/ml	0.9910	0.8631	0.6127	0.9018	N/A

% total axons			**Perpendicular**				
			200mV/mm + ToxinB				
			0 ng/ml	0.1 ng/ml	0.5 ng/ml	1 ng/ml	10 ng/ml
**Perpendicular**	200mV/mm + ToxinB	0 ng/ml	N/A				
		0.1 ng/ml	>0.9999	N/A			
		0.5 ng/ml	>0.9999	0.9992	N/A		
		1 ng/ml	0.5512	0.6570	0.9985	N/A	
		10 ng/ml	0.1267	0.2277	0.8631	>0.9999	N/A

Directedness		200mV/mm + ToxinB				
		0 ng/ml	0.1 ng/ml	0.5 ng/ml	1 ng/ml	10 ng/ml
200mV/mm + ToxinB	0 ng/ml	N/A				
	0.1 ng/ml	0.9247	N/A			
	0.5 ng/ml	0.6817	>0.9999	N/A		
	1 ng/ml	**<0.0001**	**<0.0001**	**<0.0001**	N/A	
	10 ng/ml	**<0.0001**	**0.0006**	**<0.0001**	0.8951	N/A

C

% total axons	No EF	1 ng/ml ToxinB
Cathode	34% (± 3%)	31% (± 1%)
Anode	30% (± 3%)	36% (± 4%)
Perpendicular	36% (± 1%)	33% (± 6%)
Directedness	-0.06 (± 0.03)	0.06 (± 0.02)
n experiment	3	3
n axons	121	154

D

		1ng/ml ToxinB		
		Cathode	Anode	Perpendicular
Control	Cathode	>0.9999		
	Anode		0.9991	
	Perpendicular			>0.9999
Directedness		0.1624		

Figure S5: **ToxinB partially neutralizes cathode-directed growth of RGC axons.** Explants were grown overnight in varying concentrations of ToxinB, then exposed to an EF of 200 mV/mm. ToxinB was replenished 1 hour before initiating EF exposure. (A) Average neurite directedness and percent axons growing towards the cathode, anode, or perpendicular to the EF was quantified (see METHODS). Number of experiments and total axons quantified are listed per condition. Error represent SD. (B) P-values for two-way analysis of variance performed on data in (A) with Tukey's multiple comparisons test. (C) Explants were grown overnight in 1ng/ml of ToxinB. ToxinB was replenished 1 hour before initiating time-lapsed microscopy. Average neurite directedness and percent axons growing towards the cathode, anode, or perpendicular to the EF was quantified. Number of experiments and total axons quantified are listed per condition. Error represent SD. (D) P-values for two-way analysis of variance performed on data in (C) with Tukey's multiple comparisons test.
