# Supplementary material for: Physiologic Electrical Fields Direct Retinal Ganglion Cell Axon Growth In Vitro
Source: Invest Ophthalmol Vis Sci. 2019 Aug;60(10):3659–68. doi: 10.1167/iovs.18-25118 (PMC6716951; doi:10.1167/iovs.18-25118)
Supplement: Supplement 8 [file iovs-60-10-10_s08.pdf]

Supplemental Figure 6

A

Rate of axon growth (µm/hr)	No EF		200mV/mm + ToxinB		
	0 ng/ml	1 ng/ml	0 ng/ml	1 ng/ml	10 ng/ml
Cathode	40.2 (± 7.2)	44.3 (± 8.2)	45.2 (± 7.8)	43.2 (± 3.4)	46.7 (± 9.3)
Anode	37.7 (± 7.1)	43.3 (± 5.3)	42.7 (± 4.2)	29.0 (± 7.8)	43.6 (± 4.8)
Perpendicular	39.1 (± 13.5)	44.4 (± 10.5)	39.8 (± 6.6)	30.2 (± 5.5)	43.1 (± 3.1)
n experiment	3	3	6	3	3
n axons	121	154	271	173	122

B

Rate of axon growth (µm/hr)			**Cathode**				
			No EF		200mV/mm		
			0 ng/ml	1 ng/ml	0 ng/ml	1 ng/ml	10 ng/ml
**Cathode**	No EF	0 ng/ml	N/A		0.9996	>0.9999	0.9992
		1 ng/ml	>0.9999	N/A	>0.9999	>0.9999	>0.9999
	200mV/mm	0 ng/ml			N/A		
		1 ng/ml			>0.9999	N/A	
		10 ng/ml			>0.9999	>0.9999	N/A

Rate of axon growth (µm/hr)			**Anode**				
			No EF		200mV/mm		
			0 ng/ml	1 ng/ml	0 ng/ml	1 ng/ml	10 ng/ml
**Anode**	No EF	0 ng/ml	N/A		0.9996	0.9771	>0.9999
		1 ng/ml	0.9998	N/A	>0.9999	0.5594	>0.9999
	200mV/mm	0 ng/ml			N/A		
		1 ng/ml			0.3893	N/A	
		10 ng/ml			>0.9999	0.6284	N/A

Rate of axon growth (µm/hr)			**Perpendicular**				
			No EF		200mV/mm		
			0 ng/ml	1 ng/ml	0 ng/ml	1 ng/ml	10 ng/ml
**Perpendicular**	No EF	0 ng/ml	N/A		>0.9999	0.9748	>0.9999
		1 ng/ml	0.9999	N/A	0.9999	0.5724	>0.9999
	200mV/mm	0 ng/ml			N/A		
		1 ng/ml			0.8753	N/A	
		10 ng/ml			>0.9999	0.8525	N/A

Figure S6: **ToxinB does not affect rate of RGC axon growth.** Explants were grown overnight in 1ng/ml or 10ng/ml of ToxinB, then exposed to an EF of 200 mV/mm. ToxinB was replenished 1 hour before initiating EF exposure. The effect of 1ng/ml and 10ng/ml of ToxinB on rate of axon growth was quantified and shown in (A). Number of experiments and total axons quantified are listed per condition. Error represent SD. (B) P-values for two-way analysis of variance performed on data in (A) with Tukey's multiple comparisons test.
