# Supplementary material for: Physiologic Electrical Fields Direct Retinal Ganglion Cell Axon Growth In Vitro
Source: Invest Ophthalmol Vis Sci. 2019 Aug;60(10):3659–68. doi: 10.1167/iovs.18-25118 (PMC6716951; doi:10.1167/iovs.18-25118)
Supplement: Supplement 9 [file iovs-60-10-10_s09.pdf]

Supplemental Figure 7

A

% total axons	Control	100 µM Z62954982
Cathode	66% (± 6%)	31% (± 4%)
Anode	10% (± 8%)	31% (± 3%)
Perpendicular	25% (± 1%)	39% (± 3%)
Directedness	-0.49 (± 0.13)	-0.02 (± 0.08)
n experiments	3	3
n axons	175	152

B

Rate of axon growth (µm/hr)	Control	100 µM Z62954982
Cathode	45.6 (± 15.1)	33.0 (± 6.6)
Anode	32.0 (± 8.9)	26.4 (± 13.2)
Perpendicular	40.1 (± 16.0)	33.0 (± 11.7)
n experiments	3	3
n axons	175	152

C

% total axons		100 µM Z62954982		
		Cathode	Anode	Perpendicular
Control	Cathode	**0.0216**		
	Anode		0.3630	
	Perpendicular			0.2992
	Directedness	**0.0062**		

D

Rate of axon growth (µm/hr)		100 µM Z62954982		
		Cathode	Anode	Perpendicular
Control	Cathode	0.1479		
	Anode		0.6813	
	Perpendicular			0.5261

Figure S7: Rac1 inhibitor Z62954982 neutralizes cathode-directed growth. Explants were grown overnight, then treated with 100mM Z62954982. Two hours later, cultures were exposed to an EF of 200 mV/mm. (A) The percentage of RGC axons growing towards the cathode, anode, versus perpendicular to the EF was quantified. Number of experiments and total axons quantified are listed per condition. Error represent SD. (B) Z62954982 effect on rate of axon growth was also quantified. Number of experiments and total axons

quantified are listed per condition. Error represent SD. (C-D) P-values for two-way ANOVA with Sidak's multiple comparison test of values from (A) and (B), respectively.
