# Supplementary material for: Physiologic Electrical Fields Direct Retinal Ganglion Cell Axon Growth In Vitro
Source: Invest Ophthalmol Vis Sci. 2019 Aug;60(10):3659–68. doi: 10.1167/iovs.18-25118 (PMC6716951; doi:10.1167/iovs.18-25118)
Supplement: Supplement 10 [file iovs-60-10-10_s10.pdf]

Supplemental Figure 8

A

Relative Rac1-GTP Levels

Duration of EF Exposure	0 mins	30 mins	60 mins	150 mins	240 mins	300 mins (240 mins + 60 mins without EF)
Relative Rac1-GTP Levels	100% (± 61.5%)	57.7% (± 23.2%)	53.5% (± 35.5%)	77.0% (± 34.0%)	153.6% (± 45.6%)	76.0% (± 47.8%)
n experiments	10	7	5	5	5	6

B

One-way ANOVA	0 mins	30 mins	60 mins	150 mins	240 mins	300 mins (240 mins + 60 mins without EF)
0 mins	N/A					
30 mins	0.4306	N/A				
60 mins	0.4444	>0.9999	N/A			
150 mins	0.9381	0.9777	0.9631	N/A		
240 mins	0.2920	**0.0128**	**0.0174**	0.1135	N/A	
300 mins (240 mins + 60 mins without EF)	0.9072	0.9781	0.9635	>0.9999	0.0816	N/A

Figure S8: Rac1-GTP levels increase after 4 hours of EF exposure in RGCs. Cultures of purified RGCs were grown overnight then exposed to an EF of 200 mV/mm for varying durations of time. (A) Level of Rac1-GTP relative to control cultures was quantified from cell lysates. (B) P-values for one-way ANOVA test performed on data in (A) with Tukey's multiple comparisons test.
